# Effects of high dietary inclusion of *Arthrospira platensis*, either extruded or supplemented with a super-dosing multi-enzyme mixture, on broiler growth performance and major meat quality parameters

**DOI:** 10.1186/s12917-024-04027-6

**Published:** 2024-05-06

**Authors:** Mónica M. Costa, Maria P. Spínola, Beatriz Tavares, José M. Pestana, João C. Tavares, Cátia F. Martins, Cristina M. Alfaia, Daniela F. P. Carvalho, Ana R. Mendes, Joana I. Ferreira, Miguel P. Mourato, Madalena M. Lordelo, José A. M. Prates

**Affiliations:** 1 CIISA - Centro de Investigação Interdisciplinar em Sanidade Animal, Faculdade de Medicina Veterinária, Universidade de Lisboa, Av. da Universidade Técnica, 1300-477 Lisboa, Portugal; 2 AL4AnimalS - Laboratório Associado para Ciência Animal e Veterinária, Av. da Universidade Técnica, 1300-477 Lisboa, Portugal; 3 Instituto Superior de Agronomia, Universidade de Lisboa, Tapada da Ajuda, 1349-017 Lisboa, Portugal; 4 LEAF - Linking Landscape, Environment, Agriculture and Food, Instituto Superior de Agronomia, Universidade de Lisboa, Associated Laboratory TERRA, Tapada da Ajuda, 1349-017 Lisboa, Portugal

**Keywords:** Microalga extrusion, Feed enzyme, Broiler chicken, Growth performance, Carcass trait, Meat quality

## Abstract

**Background:**

This investigation assessed the effects of high dietary inclusion of Spirulina (*Arthrospira platensis*) on broiler chicken growth performance, meat quality and nutritional attributes. For this, 120 male broiler chicks were housed in 40 battery brooders (three birds per brooder). Initially, for 14 days, a standard corn and soybean meal diet was administered. Subsequently, from days 14 to 35, chicks were assigned to one of the four dietary treatments (*n* = 10 per treatment): (1) control diet (CTR); (2) diet with 15% Spirulina (SP); (3) diet with 15% extruded Spirulina (SPE); and (4) diet with 15% Spirulina plus a super-dosing enzymes supplement (0.20% pancreatin extract and 0.01% lysozyme) (SPM).

**Results:**

Throughout the experimental period, both SP and SPM diets resulted in decreased final body weight and body weight gain compared to control (*p* < 0.001), with the SPE diet showing comparable results to CTR. The SPE diet prompted an increase in average daily feed intake (*p* = 0.026). However, all microalga treatments increased the feed conversion ratio compared to CTR. Dietary inclusion of Spirulina notably increased intestinal content viscosity (*p* < 0.010), which was mitigated by the SPM diet. Spirulina supplementation led to lower pH levels in breast meat 24 h *post-mortem* and heightened the b* colour value in both breast and thigh meats (*p* < 0.010). Furthermore, Spirulina contributed to an increased accumulation of total carotenoids, n-3 polyunsaturated fatty acids (PUFA), and saturated fatty acids (SFA), while diminishing n-6 PUFA, thus altering the n-6/n-3 and PUFA/SFA ratios favourably (*p* < 0.001). However, it also reduced zinc concentration in breast meat (*p* < 0.001).

**Conclusions:**

The findings indicate that high Spirulina levels in broiler diets impair growth due to increased intestinal viscosity, and that extrusion pre-treatment mitigates this effect. Despite reducing digesta viscosity, a super-dosing enzyme mix did not improve growth. Data also indicates that Spirulina enriches meat with antioxidants and n-3 PUFA but reduces α-tocopherol and increases saturated fats. Reduced zinc content in meat suggests the need for Spirulina biofortification to maintain its nutritional value.

## Background

The search for sustainable and alternative protein sources to replace conventional feed ingredients, like soybean meal, has intensified due to environmental and economic concerns. Microalgae have emerged as a promising alternative, drawing significant interest in their application in animal nutrition [1]. Poultry, in particular, has shown a favourable response to microalgal inclusion in feed, indicating a substantial potential for microalgae utilization in commercial feed formulations [2]. Among these, Spirulina (*Arthrospira platensis*), with its high protein content, ranging from 50 to 70%, stands out as a strong candidate to supplement or partially replace traditional protein sources in poultry diets [3–7].

Spirulina, a filamentous cyanobacterium, is renowned for its resilience in harsh environments and its complex, multicellular structure [8, 9]. Its cellular composition, reminiscent of Gram-negative bacteria, includes a robust cell wall made up of peptidoglycan and lipopolysaccharides, which presents a formidable barrier to digestion [10, 11]. Beyond its protein, Spirulina is a reservoir of carbohydrates, essential fatty acids, and a spectrum of phytonutrients, including vitamins (such as pro-vitamin A, C, and E) and minerals (iron, calcium, and zinc among others), as well as photosynthetic pigments like chlorophyll *a*, phycocyanin and various carotenoids [12–15].

However, the bioavailability of Spirulina’s nutrients, particularly proteins, is often limited by the algae’s indigestible cell wall and the stable protein-pigment complexes associated with its thylakoid membranes [16]. Advanced processing methods like extrusion have been suggested to disrupt these complexes, thereby enhancing protein digestibility [17, 18]. Enzymatic treatment with peptidases, which catalyse the hydrolysis of peptide bonds in proteins [19], has been explored as a means to improve the bioaccessibility of Spirulina proteins [17, 20]. Moreover, these treatments have the potential to modulate gut viscosity by altering protein interactions, which could impact nutrient absorption in monogastric animals like poultry [21, 22].

The use of exogenous enzymes, such as carbohydrate-active enzymes (CAZymes) and peptidases, has been recognised for its capacity to enhance the nutritional value of poultry diets [23–27]. Despite this, the specific effects of enzymes like pancreatin and lysozyme on the digestibility and nutritional uptake of Spirulina proteins in poultry remain underexplored [28]. Pancreatin, a complex mixture of digestive enzymes, and lysozyme, with its antimicrobial properties, may support the efficient digestion of Spirulina-based feeds, particularly in younger broilers with less developed digestive systems [29]. Particularly, lysozyme was selected for its ability to disrupt the Spirulina cell wall, as previously demonstrated in vitro through the release of protein, some fatty acids and chlorophyll *a* [25], and in vivo, where an increase of broiler´s digesta viscosity occurred with lysozyme supplemented Spirulina, which was suggested to be due to gelation of indigestible proteins released from algal biomass [22]. Park et al. [30] also reported an increase in dry matter and energy digestibility and a tendency for an enhancement of nitrogen digestibility in piglets fed a basal diet supplemented with lysozyme. These results were attributed to a modulation of intestinal microbiota and haematological parameters (i.e., decrease of cortisol and white blood cells) promoted by lysozyme. Abdel-Latif et al. [31] demonstrated an improvement in growth performance, nonspecific immunity and gut antioxidant status in broilers fed exogenous lysozyme. In addition, Ribeiro et al. [32] showed an increase in structural muscle protein synthesis in piglets fed Spirulina and lysozyme-containing diets, thus, reinforcing the benefits of using such dietary enzymatic supplementation.

Previous research has indicated that enzymes like those in pancreatin can facilitate dietary transitions from traditional feed ingredients to alternative sources, improving nutrient digestibility [29, 33]. The interplay between super-dosed (higher than recommended) multi-enzymes and higher levels of Spirulina in feed warrants investigation, especially considering the potential for these enzymes to improve growth performance, gut health and nutrient absorption in broilers [33–35]. Thus, this study was designed to scrutinise the effect of a 15% Spirulina inclusion in broiler diets, administered in extruded form or combined with a super-dosing enzymatic blend comprising pancreatin and lysozyme, on growth performance, gut viscosity, carcass characteristics, and the quality and nutritional profile of meat during the growth phase from day 14 to day 35.

## Results

### Growth performance and gastrointestinal tract

Table 1 shows the effect of experimental diets on broiler growth performance. The SP and SPM diets reduced (*p* < 0.001) body weight (BW) relative to control, on day 21, but there were no differences between SPE and the control. All microalga-containing diets decreased BW (*p* < 0.001) compared to control, on day 28, although the SPE diet led to intermediate BW values between those with SP and SPM and the control. A similar effect (*p* < 0.001) was noted on day 35, except for broilers fed the SPE diet, which had no differences (*p* > 0.05) in their BW compared to control animals. All microalga-containing diets reduced (*p* < 0.001) body weight gain (BWG) in comparison to the control, even though the SPE diet led to intermediate BWG values, on days 14 to 21. Similar significant results were found (*p* < 0.001) on days 21 to 28, but without differences between SPE and the control or other microalga treatments. However, no significant differences (*p* > 0.076) in BWG were observed between treatments, on days 28 to 35. The SP and SPM diets significantly reduced (*p* < 0.001) BWG compared to the control, with no differences between the SPE diet and the control, during the experimental period (days 14 to 35). Moreover, the broilers fed the SP and SPM diets had a lower (*p* = 0.001) average daily feed intake (ADFI) than those fed the control diet, but birds fed the SPE diet were not different from the control animals, on days 14 to 21. Nevertheless, no differences were found (*p* > 0.05) between microalga- and control-fed broilers for ADFI, on days 14 to 35, albeit the SPE diet increased (*p* = 0.026) this parameter compared to the SP diet. All microalga-containing diets increased feed conversion ratio (FCR) compared to control, during the experimental period (*p* < 0.001), and a tendency for this outcome occurred on days 21 to 28.

**Table 1:** Growth performance of broilers

Item	CTR^1^	SP^1^	SPE^1^	SPM^1^	SEM	*p*-value
**Body weight, g**						
day 14	368	351	382	380	10.3	0.139
day 21	672^a^	538^b^	614^a^	550^b^	15.9	< 0.001
day 28	1046^a^	843^c^	946^b^	851^c^	21.1	< 0.001
day 35	1428^a^	1183^b^	1349^a^	1194^b^	35.6	< 0.001
**Body weight gain, g/day**						
day 14–21	42.9^a^	26.6^c^	33.2^b^	26.1^c^	1.65	< 0.001
day 21–28	52.5^a^	43.6^b^	47.4^ab^	42.9^b^	1.45	< 0.001
day 28–35	54.7	48.5	57.7	49.0	2.82	0.075
day 14–35	50.2^a^	39.6^b^	46.1^a^	38.7^b^	1.43	< 0.001
**Average daily feed intake, g/pen**						
day 14–21	189^a^	159^b^	186^a^	163^b^	6.08	0.001
day 21–28	249	228	261	238	9.89	0.116
day 28–35	307	270	314	281	12.8	0.064
day 14–35	248^ab^	219^b^	254^a^	227^ab^	8.94	0.026
**Feed conversion ratio**						
day 14–21	1.48	2.11	1.92	2.32	0.353	0.389
day 21–28	1.58	1.91	1.91	2.21	0.166	0.080
day 28–35	1.96	2.00	1.88	2.03	0.103	0.774
day 14–35	1.64^b^	1.91^a^	1.88^a^	2.03^a^	0.057	< 0.001

SEM, standard error of the mean

^a.b.c^ Different superscripts within a row indicate a significant difference (*p* < 0.05)

^1^ CTR: Corn and soybean meal-based diet, in the control group; SP: 15% Spirulina; SPE: 15% extruded Spirulina; SPM: 15% Spirulina + 0.21% enzyme mixture (0.20% porcine pancreatin + 0.01% lysozyme)

Table 2 presents the effects of experimental diets on the relative weight and length of broilers’ gastrointestinal tract, as well as on gut content viscosity. A tendency for a reduction or an increase in crop weight was observed in birds fed SPE and SPM diets, respectively, compared to the control group. In addition, the duodenum tended (*p* = 0.051) to increase with SP and SPM diets. Furthermore, SPM diets manifested an increase in the duodenum (*p* = 0.026), jejunum (*p* = 0.006) and caecum (*p* = 0.042) length relative to the control. A discernible enhancement in ileum (*p* < 0.001) length was associated with all microalga-containing diets. Regarding gut content viscosity, the SP diet significantly heightened (*p* = 0.001) the viscosity of duodenum and jejunum contents compared to the control diet (7.39 *versus* 4.71 cP). Although a similar increase was noted with the SPE diet, a numerical reduction from SP to SPE diets was observed (7.39 to 6.57 cP). Interestingly, no significant differences (*p* > 0.05) were discerned between the SPM and control diets. The viscosity trend in ileum content (*p* = 0.003) paralleled that of the duodenum and jejunum, albeit without a discernible reduction in the SPE diet compared to the control.

**Table 2:** Relative weight and length of the gastrointestinal tract of broilers

Item	CTR^1^	SP^1^	SPE^1^	SPM^1^	SEM	*p*-value
**Relative weight of GI** ^2^ **tract, g/kg body weight**						
Crop	4.09^ab^	4.87^ab^	3.97^b^	5.55^a^	0.408	0.032
Gizzard	15.8	15.9	15.3	16.7	0.795	0.628
Liver	26.9	25.3	26.1	25.1	1.23	0.746
Pancreas	2.89	3.15	2.98	3.21	0.123	0.231
Proventriculus	4.88	4.75	4.98	5.26	0.238	0.498
Duodenum	6.72	7.45	6.86	7.84	0.296	0.051
Jejunum	12.4	12.1	11.0	13.1	0.574	0.099
Ileum	10.5	11.6	10.0	10.7	0.588	0.296
Caecum^3^	4.14	4.81	4.41	5.08	0.330	0.209
**Relative length of GI tract, cm/kg body weight**						
Duodenum	20.9^b^	23.8^ab^	23.5^ab^	25.4^a^	0.993	0.026
Jejunum	48.8^b^	56.4^ab^	49.6^ab^	63.3^a^	3.07	0.006
Ileum	51.6^b^	61.5^a^	60.8^a^	68.4^a^	2.02	< 0.001
Caecum	11.0^b^	11.9^ab^	11.9^ab^	13.6^a^	0.634	0.042
**Content viscosity, cP**						
Duodenum + jejunum	4.71^c^	7.39^a^	6.57^ab^	5.32^bc^	0.465	0.001
Ileum	8.21^b^	13.0^a^	14.2^a^	10.6^ab^	1.10	0.003

SEM, standard error of the mean

^a.b.c^ Different superscripts within a row indicate a significant difference (*p* < 0.05)

^1^ CTR: Corn and soybean meal-based diet, in the control group; SP: 15% Spirulina; SPE: 15% extruded Spirulina; SPM: 15% Spirulina + 0.21% enzyme mixture (0.20% porcine pancreatin + 0.01% lysozyme)

^2^ GI: Gastrointestinal

^3^ Caecum: weight of the 2 caeca

### Carcass traits and meat quality assessment

Table 3 elucidates the effects of varied dietary treatments on broiler carcass attributes and meat quality. The data unveils a distinct reduction in the pH24h values of breast meat in the groups fed with microalgal diets (SP, SPE, SPM) as opposed to the control group (*p* = 0.001). Although the lightness (L*) of both breast and thigh meats appeared unaffected by the algal supplementation in a significant manner, a slight numeric escalation and declination were observed in thigh meat with SPM and SPE diets respectively. Remarkably, the algal treatments substantially amplified the yellowness (b*) of both meat types, exhibiting a twofold increment (*p* < 0.001). This suggests a notable influence of microalgal diets on the colour profile, particularly the yellowness, of broiler meats, which might be indicative of altered pigment deposition.

**Table 3:** Carcass traits and meat quality of broilers’ breast and thigh meats fed with Spirulina

Item	CTR^1^	SP^1^	SPE^1^	SPM^1^	SEM	*p*-value	CTR^1^	SP^1^	SPE^1^	SPM^1^	SEM	*p*-value
Breast meat							Thigh meat					
pH24h	5.66^a^	5.48^b^	5.49^b^	5.45^b^	0.035	0.001	5.84	5.78	5.77	5.75	0.039	0.423
Lightness (L*)	62.1	59.5	59.7	60.8	0.81	0.106	56.1^ab^	54.6^ab^	53.7^b^	56.8^a^	0.75	0.025
Redness (a*)	4.19	4.40	3.53	3.81	0.313	0.220	6.17	7.28	7.23	6.46	0.545	0.384
Yellowness (b*)	7.59^b^	17.4^a^	17.8^a^	16.4^a^	0.642	< 0.001	10.4^b^	20.9^a^	21.5^a^	20.2^a^	0.648	< 0.001

SEM, standard error of the mean

^a.b^ Different superscripts within a row indicate a significant difference (*p* < 0.05)

^1^ CTR: Corn and soybean meal-based diet, in the control group; SP: 15% Spirulina; SPE: 15% extruded Spirulina; SPM: 15% Spirulina + 0.21% enzyme mixture (0.20% porcine pancreatin + 0.01% lysozyme)

### Oxidative stability, vitamin E homologues and total pigments of meat

Table 4 illustrates the impact of different dietary interventions on the oxidative stability of broiler breast meat, evaluated at two distinct time intervals (days 0 and 8). The data reveals no significant influence from either the diet (*p* = 0.409) or the period (*p* = 0.854) on the levels of TBA reactive substances, particularly malondialdehyde. Furthermore, the interaction between the dietary treatments and the period did not yield any significant effect (*p* = 0.246). Despite the statistical insignificance, it’s noteworthy that the malondialdehyde levels exhibited a numerical elevation, nearly tripling in value, under the SPM diet in comparison to the control, albeit with substantial variability.

**Table 4:** Oxidative stability of broiler breast meat measured as thiobarbituric acid reactive substances (TBARS)

Malondialdehyde, mg/kg	CTR^1^	SP^1^	SPE^1^	SPM^1^	*p*-value		
					Diet	Period	Diet*Period
day 0	0.31 ± 0.424	0.31 ± 0.265	0.69 ± 0.997	0.31 ± 0.240	0.409	0.854	0.246
day 8	0.24 ± 0.235	0.52 ± 0.370	0.25 ± 0.119	0.69 ± 0.387			

^1^CTR: Corn and soybean meal-based diet, in the control group; SP: 15% Spirulina; SPE: 15% extruded Spirulina; SPM: 15% Spirulina + 0.21% enzyme mixture (0.20% porcine pancreatin + 0.01% lysozyme)

Values are presented as least square means ± standard deviation

Table 5 presents the impact of dietary variations on the content of vitamin E homologues and pigments in broilers’ breast and thigh meats. All microalga-inclusive diets notably diminished the levels of α-tocopherol across both meat types, as well as the minor diterpenes, γ-tocopherol + β-tocotrienol. Additionally, the SP and SPM diets significantly curtailed the concentration of α-tocotrienol in the breast meat, with the SPE diet rendering intermediate values. However, the thigh meat demonstrated no significant variation in α-tocotrienol content across the diets. Notably, β-tocopherol remained undetectable in both meat types across all treatments.

**Table 5:** Diterpene profile and pigments of broiler meats

Item	CTR^1^	SP^1^	SPE^1^	SPM^1^	SEM	*p*-value	CTR^1^	SP^1^	SPE^1^	SPM^1^	SEM	*p*-value
Breast meat							Thigh meat					
**Diterpene profile, µg/g**												
α-Tocopherol	8.28^a^	3.36^b^	4.19^b^	3.65^b^	0.329	< 0.001	12.0^a^	6.01^b^	6.38^b^	6.26^b^	0.532	< 0.001
γ-Tocopherol + β-tocotrienol	0.13^a^	0.056^b^	0.068^b^	0.058^b^	0.0048	< 0.001	0.13^a^	0.083^b^	0.099^b^	0.082^b^	0.0063	< 0.001
α-Tocotrienol	0.20^a^	0.15^b^	0.17^ab^	0.16^b^	0.011	0.007	0.24	0.20	0.22	0.21	0.014	0.206
β-Tocopherol	n.d.	n.d.	n.d.	n.d.	n.d.	n.d.	n.d.	n.d.	n.d.	n.d.	n.d.	n.d.
**Pigments, µg/100 g**												
Chlorophyll *a*^2^	6.01	5.72	7.05	5.30	1.332	0.798	7.26^b^	7.02^b^	13.5^a^	9.19^ab^	1.704	0.020
Chlorophyll *b*^3^	10.3	10.2	13.4	8.91	2.36	0.564	14.1^ab^	12.6^b^	23.6^a^	15.6^ab^	3.060	0.039
Total chlorophylls^4^	16.3	15.9	20.4	14.2	3.67	0.654	21.4	19.6	37.0	30.0	5.05	0.058
β-Carotene	0.072	0.068	0.072	0.067	0.0047	0.825	0.070^b^	0.092^ab^	0.11^a^	0.082^ab^	0.0085	0.021
Total carotenoids^5^	27.3^b^	172^a^	165^a^	159^a^	9.6	< 0.001	35.0^b^	193^a^	186^a^	160^a^	9.9	< 0.001
Total chlorophylls and carotenoids^6^	43.6^b^	188^a^	185^a^	173^a^	11.8	< 0.001	57.6^c^	213^ab^	223^a^	190^b^	11.4	< 0.001

SEM, standard error of the mean

^a.b.c^ Different superscripts within a row indicate a significant difference (*p* < 0.05)

^1^CTR: Corn and soybean meal-based diet, in the control group; SP: 15% Spirulina; SPE: 15% extruded Spirulina; SPM: 15% Spirulina + 0.21% enzyme mixture (0.20% porcine pancreatin + 0.01% lysozyme)

^2^ Ca: Chlorophyll a = 11.24 × A662 nm − 2.04 × A645 nm

^3^ Cb: Chlorophyll b = 20.13 × A645 nm − 4.19 × A662 nm

^4^ Ca + b: Total chlorophylls = 7.05 × A662 nm + 18.09 × A645 nm

^5^ Cx + c: Total carotenoids = (1000 × A470 nm − 1.90 × Ca − 63.14 × Cb) / 214

^6^ Ccc: Total chlorophylls and carotenoids = (Ca + b) + (Cx + c)

n.d.: Not detected

The chlorophyll content showcased no significant alterations induced by the diets in the breast meat for chlorophyll *a*, *b*, and total, and in the thigh meat for chlorophyll *b*. Nonetheless, the SPE diet enhanced the quantity of chlorophyll *a* in the breast meat vis-a-vis the control, with the SPM diet rendering intermediate values for this compound.

Regarding β-carotene, there was no discernible impact on its concentration in the breast meat across the dietary treatments. However, in the thigh meat, the SPE diet elevated β-carotene levels compared to the control, with SP and SPM diets delivering intermediate values. The microalga treatments significantly augmented the total carotenoid content in both meat types, with a remarkable 6-fold and 5-fold increase in the breast and thigh meats respectively. This considerable augmentation in carotenoid content consequently propelled an increase in the summative values of total chlorophylls and carotenoids in both meat types, underscoring the significant influence of dietary microalga incorporation on the diterpene and pigment profiles in broiler meats.

### Total lipids, fatty acid composition and health-related lipid indices of meat

Table 6 shows the effects of varying dietary regimens on the lipid content, fatty acid composition and health-related lipid indices in the meat of broilers. In the breast meat, it was observed that SP and SPM diets significantly reduced the total lipid concentration (*p* = 0.009), with a similar trend, although not statistically significant, noticed in the SPE diet. No significant impact on cholesterol levels was noted across all diets (*p* > 0.05). The inclusion of microalgae in diets revealed a noteworthy increase in certain saturated fatty acids (SFA) such as 14:0, 16:0, and 17:0 (*p* < 0.001), alongside monounsaturated fatty acids (MUFA) like 16:1c9 and 18:1c11 (*p* < 0.001), and polyunsaturated fatty acids (PUFA) including 18:3n-3, 20:3n-6, 20:3n-3, 20:5n-3, 22:5n-3, and 22:6n-3 (*p* < 0.010). Notably, the SPE diet significantly escalated the levels of 14:1c9 (*p* = 0.001), with the SPM diet showing intermediate values for this minor fatty acid compared to the control and SPE diets. An elevation in the content of 15:0 was linked to the SPM diet (*p* = 0.028), presenting intermediate values with SP and SPE diets. Broilers on SP and SPE diets exhibited higher levels of 16:1c7 (*p* = 0.005) and 17:1c9 (*p* < 0.001) in breast meat compared to the control, with a tendency of increased 16:1c7 observed in the SPM diet. Additionally, the SP diet significantly enhanced the levels of 20:1c11 (*p* = 0.047) and 20:4n-6 (*p* = 0.040) relative to control, with intermediate values noted in SPE and SPM diets. On the flip side, a reduction in 18:2n-6 and 18:3n-6 was associated with alga treatments (*p* < 0.001). Microalga inclusion contributed to an increment in total SFA and n-3 PUFA, albeit the SPE diet showcased higher n-3 PUFA values compared to SP and SPM diets. Moreover, a decline in total PUFA, n-6 PUFA, PUFA/SFA, and n-6/n-3 ratios was evident in alga treatments (*p* < 0.001), with a pronounced reduction in the n-6/n-3 ratio in the SPE diet.

**Table 6:** Total lipid content, cholesterol content and fatty acid profile of broiler meats

Item	CTR^1^	SP^1^	SPE^1^	SPM^1^	SEM	*p*-value	CTR^1^	SP^1^	SPE^1^	SPM^1^	SEM	*p*-value
	Breast meat						Thigh meat					
**Total lipids, g/100 g**	1.59^a^	1.10^b^	1.19^ab^	1.04^b^	0.115	0.009	3.09	2.96	3.35	2.92	0.224	0.523
**Cholesterol, mg/g**	0.59	0.50	0.56	0.59	0.034	0.222	0.65	0.65	0.71	0.70	0.041	0.582
**Fatty acid composition, g/100 g FA**												
12:0	0.023	0.031	0.029	0.023	0.005	0.488	0.021	0.024	0.026	0.024	0.0014	0.129
14:0	0.301^b^	0.382^a^	0.394^a^	0.374^a^	0.013	< 0.001	0.34^b^	0.48^a^	0.47^a^	0.47^a^	0.010	< 0.001
14:1c9	0.031^b^	0.050^b^	0.077^a^	0.057^ab^	0.007	0.001	0.049^b^	0.090^a^	0.104^a^	0.090^a^	0.0048	< 0.001
15:0	0.081^b^	0.094^ab^	0.094^ab^	0.099^a^	0.004	0.028	0.073^b^	0.103^a^	0.092^a^	0.103^a^	0.0039	< 0.001
16:0	15.5^b^	20.7^a^	20.9^a^	20.3^a^	0.220	< 0.001	15.4^b^	20.6^a^	20.6^a^	20.4^a^	0.27	< 0.001
16:1c7	0.425^b^	0.528^a^	0.545^a^	0.515^ab^	0.024	0.005	0.46^b^	0.63^a^	0.63^a^	0.61^a^	0.020	< 0.001
16:1c9	1.23^b^	2.30^a^	2.76^a^	2.27^a^	0.171	< 0.001	1.84^b^	3.43^a^	3.92^a^	3.35^a^	0.170	< 0.001
17:0	0.154^c^	0.242^a^	0.206^b^	0.261^a^	0.009	< 0.001	0.14^c^	0.23^ab^	0.20^b^	0.25^a^	0.009	< 0.001
17:1c9	0.008^b^	0.037^a^	0.047^a^	0.015^b^	0.005	< 0.001	0.000^c^	0.024^b^	0.043^a^	0.020^b^	0.0023	< 0.001
18:0	8.83	9.07	8.64	9.43	0.259	0.184	7.50	7.53	7.17	7.91	0.207	0.111
18:1c9	29.7	27.9	29.5	28.1	0.705	0.173	31.6^b^	32.9^ab^	34.0^a^	33.7^a^	0.51	0.009
18:1c11	1.48^b^	2.32^a^	2.29^a^	2.24^a^	0.089	< 0.001	1.19^b^	1.80^a^	1.76^a^	1.76^a^	0.040	< 0.001
18:2n-6	32.3^a^	22.7^b^	21.6^b^	22.2^b^	0.603	< 0.001	34.3^a^	24.2^b^	23.1^b^	23.7^b^	0.57	< 0.001
18:3n-6	0.087^a^	0.062^b^	0.049^b^	0.055^b^	0.005	< 0.001	0.094^a^	0.066^b^	0.065^b^	0.071^b^	0.0034	< 0.001
18:3n-3	0.466^c^	0.629^b^	0.791^a^	0.634^b^	0.036	< 0.001	0.53^c^	0.77^b^	0.95^a^	0.71^b^	0.029	< 0.001
20:0	0.095	0.097	0.096	0.102	0.004	0.513	0.093	0.092	0.092	0.094	0.0029	0.967
20:1c11	0.224^b^	0.272^a^	0.245^ab^	0.261^ab^	0.012	0.047	0.24	0.26	0.27	0.28	0.014	0.180
20:2n-6	0.434	0.413	0.365	0.430	0.029	0.315	0.29^a^	0.23^b^	0.20^b^	0.21^b^	0.014	< 0.001
20:3n-6	0.626^b^	0.984^a^	0.973^a^	0.948^a^	0.049	< 0.001	0.46	0.51	0.54	0.49	0.033	0.371
20:4n-6	4.22^b^	5.68^a^	5.04^ab^	5.54^ab^	0.377	0.040	2.22	2.21	2.19	2.08	0.220	0.969
20:3n-3	0.007^b^	0.029^a^	0.030^a^	0.027^a^	0.005	0.008	0.016	0.018	0.020	0.017	0.0017	0.357
20:5n-3	0.022^c^	0.099^ab^	0.125^a^	0.095^b^	0.007	< 0.001	0.017^c^	0.046^b^	0.062^a^	0.040^b^	0.0042	< 0.001
22:0	0.054	0.070	0.075	0.078	0.008	0.150	0.050^a^	0.038^b^	0.036^b^	0.040^b^	0.0021	< 0.001
22:1n-9	0.010	0.022	0.022	0.012	0.005	0.160	0.022	0.033	0.023	0.036	0.0063	0.269
22:2–6	n.d.	n.d.	n.d.	n.d.	n.d.	n.d.	0.043^b^	0.066^a^	0.060^a^	0.062^a^	0.0042	0.002
22:5n-3	0.204^b^	0.474^a^	0.532^a^	0.480^a^	0.037	0.008	0.13^c^	0.22^ab^	0.24^a^	0.18^bc^	0.015	< 0.001
22:6n-3	0.123^b^	0.281^a^	0.282^a^	0.257^a^	0.023	< 0.001	0.072^b^	0.136^a^	0.126^a^	0.125^a^	0.0126	0.004
23:0	0.092	0.121	0.128	0.147	0.011	0.404	0.046	0.042	0.040	0.045	0.0036	0.717
Others	3.27^b^	4.35^a^	4.18^ab^	5.09^a^	0.258	< 0.001	2.71	3.25	2.96	3.06	0.230	0.420
**Partial sums of fatty acids**												
SFA	25.2^b^	30.8^a^	30.6^a^	30.9^a^	0.279	< 0.001	23.7^b^	29.1^a^	28.7^a^	29.3^a^	0.33	< 0.001
MUFA	33.1	33.4	35.5	33.5	0.852	0.189	35.4^b^	39.2^a^	40.8^a^	39.9^a^	0.68	< 0.001
PUFA	38.5^a^	31.3^b^	29.6^b^	30.5^b^	0.762	< 0.001	38.2^a^	28.4^b^	27.6^b^	27.7^b^	0.72	< 0.001
n-6 PUFA	37.7^a^	29.9^b^	28.0^b^	29.1^b^	0.735	< 0.001	37.4^a^	27.3^b^	26.2^b^	26.6^b^	0.69	< 0.001
n-3 PUFA	0.789^c^	1.36^b^	1.61^a^	1.38^b^	0.039	< 0.001	0.76^c^	1.18^b^	1.40^a^	1.08^b^	0.042	< 0.001
**Nutritional ratios**												
n-6/n-3	47.9^a^	22.1^b^	17.5^c^	21.2^b^	0.516	< 0.001	49.3^a^	23.2^b^	18.7^c^	24.9^b^	0.57	< 0.001
PUFA/SFA	1.54^a^	1.01^b^	0.970^b^	0.989^b^	0.033	< 0.001	1.62^a^	0.98^b^	0.96^b^	0.95^b^	0.037	< 0.001
Health-related indices												
AI	0.234^b^	0.343^a^	0.344^a^	0.341^a^	0.0040	< 0.001	0.228^b^	0.333^a^	0.329^a^	0.330^a^	0.0050	< 0.001
TI	0.651^b^	0.831^a^	0.806^a^	0.842^a^	0.0110	< 0.001	0.601^b^	0.777^a^	0.748^a^	0.788^a^	0.0130	< 0.001
PI	55.2	55.8	52.9	54.5	2.02	0.757	48.0^a^	39.7^b^	39.3^b^	38.2^b^	1.36	< 0.001

SEM, standard error of the mean

^a.b.c^ Different superscripts within a row indicate a significant difference (*p* < 0.05)

^1^ CTR: Corn and soybean meal-based diet, in the control group; SP: 15% Spirulina; SPE: 15% extruded Spirulina; SPM: 15% Spirulina + 0.21% enzyme mixture (0.20% porcine pancreatin + 0.01% lysozyme)

n.d.: not detected

SFA = 12:0 + 14:0 + 15:0 + 16:0 + 17:0 + 18:0 + 20:0 + 22:0 + 23:0

MUFA = 14:1c9 + 16:1c7 + 16:1c9 + 17:1c9 + 18:1c9 + 18:1c11 + 20:1c11 + 22:1n-9

PUFA = 18:2n-6 + 18:3n-6 + 18:3n-3 + 20:2n-6 + 20:3n-6 + 20:4n-6 + 20:3n-3 + 20:5n-3 + 22:5n-3 + 22:6n-3

n-3 PUFA = 18:3n-3 + 20:3n-3 + 20:5n-3 + 22:5n-3 + 22:6n-3

n-6 PUFA = 18:2n-6 + 18:3n-6 + 20:2n-6 + 20:3n-6 + 20:4n-6.

AI = [C 12:0 + (4 × C 14:0) + C 16:0]/Σ UFA Ulbricht & Southgate, 1991; TI = (C 14:0 + C 16:0 + C 18:0)/[((0.5 × ΣMUFA) + (0.5 × ΣFAω6) + (3 × ΣFAω3))+ ( ΣFAω3/ ΣFAω6)] Ulbricht & Southgate, 1991; PI = (monoenoic acid × 0.025) + (dienoic acid × 1) + (trienoic acid × 2) + (tetraenoic acid × 4) + (pentanoic acid × 6) + (hexanoic acid × 8) Erickson, 1992

Concerning thigh meat, no significant alterations were found in total lipids and cholesterol (*p* > 0.05) among the treatments. Nevertheless, all microalga-containing diets elevated certain SFA (14:0, 15:0, 16:0, and 17:0) (*p* < 0.001), MUFA (14:1c9, 16:1c7, 16:1c9, 17:1c9, and 18:1c11) (*p* < 0.001), and PUFA (18:3n-3, 20:5n-3, and 22:6n-3) (*p* < 0.010). Both SPE and SPM diets significantly heightened 18:1c9 levels (*p* = 0.009) relative to the control, with the SP diet showing intermediate values. Broilers on SP and SPE diets manifested higher (*p* < 0.001) amounts of 22:5n-3 in their meat compared to the control group. All alga treatments led to an augmentation in total SFA, MUFA, and n-3 PUFA (*p* < 0.001) compared to the control, with the SPE diet yielding a higher percentage of n-3 PUFA, and intermediate values observed in SP and SPM diets. Similar to the breast meat, a reduction in total PUFA, n-6 PUFA, n-6/n-3, and PUFA/SFA ratios was noted (*p* < 0.001) in microalga-containing diets, with a more discernible effect on the n-6/n-3 ratio observed in the SPE diet. In the breast and thigh meats, both atherogenic (AI) and thrombogenic (TI) indices were significantly increased with all microalga-containing diets compared to the control group (*p* < 0.001). The peroxidizability index (PI) did not differ between treatments in the breast meat (*p* = 0.757) but was significantly decreased in thigh meat when the microalga was incorporated into the diets (*p* < 0.001).

### Mineral composition of meat

The mineral composition of broiler meats under various dietary regimens is shown in Table 7. In the context of breast meat, the data signifies no notable variances (*p* > 0.05) among the treatments concerning individual and cumulative macrominerals, and the total mineral content. However, a discernible reduction in zinc levels (*p* < 0.001) was observed with the incorporation of microalga-containing diets. Particularly, the SP and SPM diets contributed to a decline (*p* = 0.002) in the overall micromineral content when compared to the control, with the SPE diet exhibiting an intermediate value.

**Table 7:** Mineral composition of broiler meats fed with Spirulina

Item	CTR^1^	SP^1^	SPE^1^	SPM^1^	SEM	*p*-value	CTR^1^	SP^1^	SPE^1^	SPM^1^	SEM	*p*-value
Breast meat							Thigh meat					
**Macrominerals (mg/100 g fresh weight)**												
Calcium (Ca)	17.4	18.4	18.5	17.9	0.87	0.790	17.3	20.9	19.3	18.8	1.09	0.151
Magnesium (Mg)	29.1	29.6	29.7	29.4	0.66	0.924	23.9	23.8	23.7	23.6	0.38	0.964
Phosphorous (P)	267	297	270	293	11.3	0.147	244	237	239	237	4.4	0.585
Potassium (K)	389	402	406	403	6.0	0.212	364	363	363	363	3.1	0.994
Sodium (Na)	143	134	139	137	3.7	0.421	141	156	154	150	4.4	0.105
Sulphur (S)	215	212	205	210	3.2	0.154	217	216	216	213	4.1	0.893
Total	1060	1094	1068	1091	15.9	0.365	1008	1018	1015	1006	10.3	0.837
**Microminerals (mg/100 g fresh weight)**												
Copper (Cu)	0.092	0.096	0.106	0.106	0.0064	0.293	0.107^b^	0.122^a^	0.122^a^	0.118^ab^	0.0037	0.021
Iron (Fe)	1.17	1.07	1.20	1.11	0.049	0.241	1.33	1.42	1.39	1.32	0.041	0.282
Manganese (Mn)	0.040	0.037	0.039	0.038	0.0014	0.494	0.042	0.045	0.043	0.043	0.0016	0.649
Zinc (Zn)	1.04^a^	0.68^b^	0.71^b^	0.68^b^	0.041	< 0.001	1.84	1.63	1.66	1.62	0.061	0.061
Total	2.34^a^	1.89^b^	2.06^ab^	1.93^b^	0.082	0.002	3.32	3.22	3.21	3.11	0.076	0.300
Total macro- and microminerals	1063	1096	1070	1092	16.0	0.377	1012	1021	1018	1009	10.4	0.835

SEM, standard error of the mean

^a.b^ Different superscripts within a row indicate a significant difference (*p* < 0.05)

^1^ CTR: Corn and soybean meal-based diet, in the control group; SP: 15% Spirulina; SPE: 15% extruded Spirulina; SPM: 15% Spirulina + 0.21% enzyme mixture (0.20% porcine pancreatin + 0.01% lysozyme)

Transitioning to thigh meat, the dietary inclusion of microalga did not impart any significant effect (*p* > 0.05) on the macromineral composition. On the other hand, a marginal elevation in copper levels (*p* = 0.021) was noticed with SP and SPE diets, albeit with a numerically minor disparity (0.11 to 0.12 mg/100 g fresh weight) in comparison to the control diet, while the SPM diet yielded intermediate values. Moreover, a tendency towards reduced zinc content (*p* = 0.061) was associated with the alga treatments when juxtaposed with the control diet.

This description underscores the nuanced influence of microalga-containing diets on the mineral composition of broiler meats, indicating a potential zinc reduction alongside a slight copper increment in thigh meat. The findings enrich the understanding of dietary impacts on mineral profiles, aiding in formulating of nutritionally optimized feed strategies for broilers.

## Discussion

The present study elucidates the significant influence of a 15% inclusion of *A. platensis* on the growth metrics of broilers, where a marked decline in BW and BWG was observed, accompanied by a trend towards a higher FCR. Nonetheless, the application of an extrusion pre-treatment to *A. platensis* revealed a notable counteraction to this negative trend, enhancing growth performance indicators, albeit not reversing the elevated FCR. This positive shift is linked to an increased ADFI when extruded *A. platensis* was introduced, although it did not exceed the intake observed with the control diet.

The underlying mechanism for this enhancement is likely rooted in the extrusion process itself. Extrusion, which exposes *A. platensis* to intense pressure and heat, is believed to disrupt the microalga’s cell wall, potentially increasing the bioavailability of critical proteins, such as phycocyanins. The enhanced bioavailability is hypothesized to facilitate more efficient hydrolysis by the broilers’ endogenous digestive enzymes, thus improving the absorption and subsequent utilization of the nutrients contained within these proteins. Evidence supporting this hypothesis can be found in the research by Safi et al. [36], which demonstrated that high-pressure treatment disrupts the *A. platensis* cell wall, increasing the accessibility of algal proteins for extraction and absorption. Moreover, the extrusion treatment may lead to a modification of the protein structure, making the otherwise complex protein assemblies more digestible and available to the broilers’ enzymatic processes. Consequently, this could translate into an improvement of growth performance outcomes, highlighting the potential of extrusion pre-treatment as a strategy to enhance the nutritional value of *A. platensis* in poultry diets. However, while extrusion appears to mitigate the growth-inhibiting effects of *A. platensis* inclusion, further research is warranted to optimize this pre-treatment process and fully understand its implications for broiler nutrition and feed efficiency.

Recent in vitro studies have provided additional clarity on the enzymatic hydrolysis of *A. platensis* proteins following extrusion. The research by Costa et al. [17] has been particularly informative, demonstrating an enhanced breakdown of protein fractions within the 18 to 26 kDa range, identified as phycocyanin subunits [16], when extruded *A. platensis* is combined with commercial pancreatin. This finding is pivotal as it indicates that extrusion pre-treatment can lead to a more efficient enzymatic release of phycocyanin, a protein known for its nutritional and functional benefits.

However, a conundrum arises from these studies where extrusion, despite its benefits, also causes protein denaturation and aggregation. Costa et al. [17] and Spínola et al. [18] observed that these denatured proteins were often not detectable in the supernatant post-extrusion, suggesting a profound modification of their physical state. Such changes are attributed to the harsh conditions of high pressure and temperature inherent to the extrusion process, as documented in the structural studies of proteins by Ahmed et al. [37] and Buecker et al. [38]. This process typically involves an initial reversible unfolding of protein structures, which can then lead to irreversible aggregation. The implications of this structural reconfiguration for the digestibility of *A. platensis* proteins are intriguing and warrant further investigation. It is hypothesized that denaturation may expose previously inaccessible peptide bonds to enzymatic attack, thereby facilitating hydrolysis. This could mean that, while extrusion alters the structural integrity of proteins, it simultaneously enhances their susceptibility to digestive enzymes, potentially increasing the nutritional value of the proteins upon ingestion by broilers. Such a hypothesis aligns with the work of Carbonaro et al. [39], who discussed how the unfolding of protein structures could expose hydrolysis sites and thereby improve digestibility. The intersection of protein structure, digestibility and nutritional bioavailability remains a complex and dynamic area of research. The evidence points to a delicate balance between the beneficial and detrimental effects of extrusion on *A. platensis* proteins. Thus, a comprehensive understanding of this balance is essential for optimizing the use of extrusion in preparing microalgae as a feed ingredient, to enhance its utilization in poultry diets without compromising the integrity of valuable protein components.

The current study aligns with the findings of Pestana et al. [22], which revealed negative impacts on broiler growth performance when diets were supplemented with 15% *A. platensis*. This reduction in performance is attributed to the gelation of microalgal proteins, leading to an increase in digesta viscosity that impedes the action of digestive enzymes and the subsequent absorption of nutrients. In an attempt to counteract this, our study incorporated pancreatin at a concentration of 2000 mg/kg, a dosage that is double that of previously reported super-dosing levels [33–35], to hydrolyse the algal proteins and, thus, prevent gelation. This approach is supported by the work of Asare et al. [35] and Madigan-Stretton et al. [33], who observed enhancements in gut morphology, microbial flora and nutrient absorption with pancreatin or enzyme blend supplementation at lower doses. Similarly, Bromfield and Hoffman [34] reported improvements in feed conversion ratios with the use of enzymatic blends. The present study also underscores the benefits of a multi-enzyme regimen comprising both peptidases and carbohydrases, which not only prevents protein putrefaction in the caecum but also provides fermentable substrates for caecal bacteria, thus reducing intestinal viscosity and improving protein digestibility [40, 41].

Notably, the addition of lysozyme and pancreatin effectively reduced the elevated viscosity in the duodenum, jejunum and ileum observed with the Spirulina-supplemented diet. This suggests that these enzymes played a role in disrupting peptidoglycans in the cell wall and the hydrolysis of microalgal proteins. The enzymes also appeared to influence intestinal morphological changes, with the SPM diet resulting in a significant elongation of the duodenum, jejunum and caecum, an effect that was less pronounced with the SP diet alone. Such intestinal lengthening has been documented in broilers fed high levels of *A. platensis* [22] or wheat [42], typically correlated with increased digesta viscosity.

Interestingly, enzymatic treatment with pancreatin and lysozyme reduced digesta viscosity and increased intestinal length, suggesting an enhanced absorptive surface for nutrients. Indeed, previous studies showed an increase in intestinal villi length and crypts depth [31] and stimulation of dry matter and energy digestibility [30] in broilers and piglets fed a commercial diet supplemented with lysozyme. However, it did not fully mitigate the negative effects on growth performance associated with high levels of *A. platensis* in the diet. This indicates that although enzyme supplementation is beneficial, it may not be sufficient on its own to overcome the challenges posed by high levels of *A. platensis*. Therefore, it could be inferred that a combination of microalgal pre-treatment, such as extrusion to alter protein structure and facilitate digestion, with enzymatic supplementation might be necessary to fully harness the nutritional potential of *A. platensis* in broiler diets.

In the realm of meat quality, the integration of Spirulina in broiler diets has notably augmented the b* value, which is an indicator of yellowness, in both breast and thigh meat. This observation corroborates previous research by Toyomizu et al. [43], Altmann et al. [44]. and Pestana et al. [22], who reported similar findings with varying levels of *A. platensis* supplementation. The enhanced yellowness is predominantly attributed to the deposition of algal carotenoids, particularly zeaxanthin, in the muscle tissues, as evidenced by the significant increase in total carotenoids in the meat of broilers fed with microalga-enriched diets. The implications of these findings extend beyond the biochemistry of meat pigmentation to consumer perception and marketability, given that meat colour is a critical quality attribute influencing consumer preferences [45]. While pale skin is generally favoured in Europe [46], preferences in Mexico [47] and, to a varying degree, in the USA [46, 48], lean towards a yellow-orange hue, historically associated with the health of the bird [46]. This preference shift underscores the need to balance consumer perceptions, which may lean towards more pigmented meat, with the potential health benefits of carotenoid-rich, yellow-coloured meat. Carotenoids are well-recognized for their antioxidant and anti-inflammatory properties, engaging in cellular-level reactive oxygen species scavenging and modulating oxidative stress pathways [49].

Nevertheless, the present study also notes a reduction in α-tocopherol levels in the meat, which is attributed to a lower content of this vitamin in the microalga-supplemented diet compared to the control. This mirrors the findings of Pestana et al. [22] and highlights the variable vitamin E content in *A. platensis*, which is contingent on the growth conditions of the microalgae [50]. The levels of α-tocopherol found in the non-extruded and extruded *A. platensis* in this study were lower than those documented in the literature [50]. While the diminished α-tocopherol content warrants attention, it is essential to consider the broader nutritional context, as evidenced by studies such as the one by Taalab et al. [51], which indicated that carotenoids like β-carotene, derived from Spirulina, could have a more pronounced effect than vitamin E on growth performance and various haemato-biochemical and immune-oxidative stress markers in broilers. Thus, while Spirulina supplementation enhances meat pigmentation and offers potential health benefits through its carotenoid content, the balance of nutrients, particularly the interplay between carotenoids and vitamin E, remains a crucial factor for optimizing both the quality of broiler meat and its acceptance among diverse consumer bases.

The inclusion of *A. platensis* in broiler diets did not significantly influence the oxidative stability of meat, as measured by thiobarbituric acid reactive substances (TBARS), aligning with prior studies [22, 44, 52]. The unchanged TBARS levels, despite a reduction in PUFA known for their susceptibility to oxidation [53], alongside an increase in antioxidant pigments from the microalga, suggest a complex interplay where the potential oxidative effects of reduced PUFAs may be offset by the antioxidant capacity of the pigments. The study posits that the low lipid content in broiler meat could contribute to the minimal impact of *A. platensis* on TBARS values, as the breast and thigh meats are characterized by relatively low-fat percentages, thus classifying them as lean meats by established standards [54]. This leanness may inherently confer a degree of resistance to lipid peroxidation, thereby maintaining oxidative stability despite dietary variations. It is also noteworthy to consider the observed variability in TBARS values across experimental diets. Such variability underscores the complexity of factors influencing oxidative stability in meat, which may include genetic differences among broilers, variances in meat storage and handling, and subtle differences in dietary composition beyond the presence of *A. platensis*. This suggests that while *A. platensis* does not detrimentally affect meat oxidative stability, further research could elucidate the nuances of how various dietary components interact to influence TBARS levels and, by extension, meat quality.

The observed decrease in total PUFA, particularly n-6 PUFA, within the present study is attributed primarily to the reduced levels of linoleic acid (18:2 n-6) in the meat. This observation aligns with previous findings [22] and is thought to be due to the metabolic conversion of linoleic acid into longer-chain n-6 PUFAs, such as arachidonic acid (20:4n-6), a process involving enzymatic elongation and desaturation. Notably, while this transformation did manifest in the breast meat with the SP diet, it was not as evident in other dietary treatments. The competition between n-6 and n-3 fatty acid pathways is well-documented [55], where α-linolenic acid (18:3n-3) is proposed to more effectively inhibit the formation of n-6 long-chain PUFAs than linoleic acid inhibits the formation of n-3 long-chain PUFAs. The study noted an increase in n-3 PUFA, including eicosapentaenoic acid (EPA), docosahexaenoic acid (DHA) and docosapentaenoic acid (DPA), which was unexpected given their absence in the experimental diets. This points to the in vivo conversion of α-linolenic acid to these longer-chain derivatives, albeit the conversion is recognized as generally inefficient. The impact of extrusion on the bioavailability of these fatty acids is underscored by the enhanced levels of n-3 PUFA in meats from the SPE diet. The health benefits of n-3 PUFAs, especially EPA and DHA, are linked to their anti-inflammatory properties [56, 57], and the present study observed a favourable decrease in the n-6/n-3 PUFA ratio, particularly with the SPE diet. This is beneficial despite the ratios not meeting the ideal of less than 4, as suggested by Wood et al. [58], to balance inflammatory and anti-inflammatory effects. Conversely, the study also found an increase in SFA, predominantly palmitic acid (16:0), which can have negative health implications by elevating low-density lipoprotein cholesterol levels and potentially increasing cardiovascular disease risk [59]. However, the PUFA/SFA ratios remained above the recommended minimum of 0.49, which is considered beneficial for cardiovascular health, with only the control diet meeting the more stringent ratio recommendations of 1.4 to 2.4 [60–62]. This multifaceted interaction among dietary fatty acids emphasizes the complexity of nutrition and health implications. While *A. platensis* incorporation did alter fatty acid profiles in broiler meat, the broader implications for human consumption and health require consideration of not only the PUFA/SFA ratios but also the specific types of fatty acids present and their known health effects. Moreover, the significant increase of AI and TI values in the meat with the microalga is a consequence of the enhancement of SFA together with a decrease of n-6 PUFA. The most atherogenic fatty acids reported are 12:0 and 14:0 [63], and, in the present study, 14:0 was increased in the breast and thigh meat with the microalga. The additional enhancement of 16:0 with *A. platensis*, which was the major fatty acid in meat, contributed to the increase of both indices. Indeed, 16:0 was previously reported as being responsible for raising the levels of LDL-cholesterol [59], and, thus, might be considered atherogenic. The increase of n-3 PUFA with the presence of microalga was not enough to have a major impact on the indices, because these fatty acids were found in small amounts (< 2.0%) in the meat. Altogether, these results could indicate a deleterious effect of meat derived from *A. platensis-*containing treatments, namely an increased predisposition to cardiovascular diseases caused by platelet aggregation [64, 65], if the AI and TI levels were not lower or slightly higher than those required for a healthy diet, i.e. 1.0 and 0.5 for AI and TI, respectively [66]. The AI and TI levels were similar to those found in the breast meat of broilers fed a commercial basal diet [67]. The present findings can be attributed to the high levels of *A. platensis*, since previous studies showed no significant effect on such indices in the egg yolk of laying hens feeding a diet with up to 3% of *A. platensis* [68], or in the serum of broiler chickens drinking up to 20 g of microalga /L [69]. In addition, the peroxidizability index decreased with microalga treatments in the thigh meat, which was due to a reduction of n-6 PUFA, and, consequently, total PUFA. This indicates that the meat from animals fed *A. platensis* was probably less prone to peroxidation, although that was not reflected in the TBARS values.

The observation that dietary Spirulina can cause a reduction in the zinc content of broiler meat, as evidenced in both breast and thigh tissues, presents a significant nutritional implication. The lower zinc content in breast meat from diets incorporating *A. platensis* is particularly noteworthy, given zinc’s crucial biological roles. Zinc is integral to a myriad of physiological functions, serving as an essential element for numerous enzymes and transcription factors. It facilitates vital processes such as nucleic acid synthesis and cellular division [70]. Additionally, zinc possesses antioxidant properties, which confer anti-inflammatory benefits [71]. Given these considerations, the adequacy of zinc intake from poultry meat becomes a matter of concern. To meet the recommended dietary intake of zinc for adults, substantial portions of meat would be required, a demand that may not align with typical consumption patterns [72]. This nutritional shortfall underscores the necessity for innovative strategies to enhance the zinc content of broiler meat. Biofortification of *A. platensis* with zinc could represent a viable solution to this challenge. Such a strategy is beneficial in other monogastric animals, like growing rabbits, where it improves growth performance, nutrient digestibility, and antioxidant status [73]. The enhancement of *A. platensis* with zinc could, therefore, be a promising avenue for research and application. This approach could not only rectify the diminished zinc content in broiler meat attributed to Spirulina supplementation but also leverage the microalgae’s potential as a nutrient-dense feed ingredient. Integrating zinc enrichment into the production of *A. platensis* may yield a dual benefit: improving the mineral nutrition of poultry and, by extension, enhancing the nutritional quality of poultry products for human consumption. Further investigations into the biofortification of *A. platensis* are warranted to explore this potential and to develop strategies that could lead to a more nutrient-rich profile in broiler meat.

## Conclusion

This study offers a comprehensive examination of the implications of incorporating *A. platensis* at a 15% inclusion rate in broiler diets. The detrimental impact on growth performance observed with this dietary regime suggests that such high levels of Spirulina may not be advantageous without appropriate pre-treatment. The application of extrusion as a pre-treatment method alleviated these adverse effects, likely by enhancing the bioavailability of proteins for digestion. Despite these improvements, extrusion did not effectively reduce intestinal content viscosity, indicating that further processing advancements are required to prevent the formation of unpalatable protein structures.

Enzymatic intervention with a super-dosing multi-enzyme mixture demonstrated efficacy in reducing digesta viscosity, underscoring the potential of enzymatic treatments in improving the digestibility of Spirulina-based feeds. The nutritional benefits of dietary Spirulina, evidenced by the increased presence of antioxidant carotenoids and essential n-3 fatty acids, align with current health trends favouring foods that support anti-inflammatory responses and overall health. Conversely, the decrease in α-tocopherol levels and the increase in saturated fatty acids present potential challenges that warrant attention.

Finally, the observed decrease in zinc content with the incorporation of Spirulina in the diet, particularly in breast meat, poses a concern for nutritional completeness and invites consideration of zinc biofortification in future Spirulina cultivation.

Looking ahead, the findings advocate for further research into combined mechanical and enzymatic pre-treatments of Spirulina to optimize its use in broiler diets. Such studies should aim to refine growth performance, nutrient digestibility and meat quality. Additionally, the exploration of novel peptidases tailored for Spirulina protein hydrolysis offers a promising area for improving the nutritional value of broiler feeds. This work underscores the importance of continuous innovation in feed technology to enhance the sustainability and nutritional outcomes of poultry production systems.

## Methods

### Animal welfare declaration

The experimental methodologies employed in this study abided by the requisite ethical norms and regulations. The procedural blueprint received approval from the Ethics Commission of CIISA/FMV (Centre for Interdisciplinary Research in Animal Health, Faculty of Veterinary Medicine) and the Animal Care Committee of the National Veterinary Authority (Direção Geral de Alimentação e Veterinária, Lisbon, Portugal). Moreover, this study conformed to the stipulations and specific guidelines encapsulated in the European Union legislation [74] regarding the employment of animals in scientific investigations. The Animal Welfare Committee of the School of Agriculture at the University of Lisbon (ORBEA/ISA) further sanctioned the experimental methodologies involving animals, allocating the study a protocol code number 0421/000/000/2022.

### Animals, management and dietary treatments

A cohort of 120 one-day-old male Ross 308 broiler chicks, acquired from Pinto Valouro (Bombarral, Portugal), with an initial body weight of 39.3 ± 2.30 g, were accommodated in 40 wire-floored enclosures for 35 days, adhering to the methodologies elucidated in Alfaia et al. [75], Pestana et al. [22] and Costa et al. [76]. These avian subjects were nurtured in a climate-controlled chamber, observing standard brooding protocols inclusive of optimal lighting conditions, i.e. a 24-hour light cycle. On the inception day, the ambient temperature was stabilized at 31 °C, transitioning to 30 °C on the subsequent day. From day 3 to 27, a gradual temperature decrement of 1 °C every 3 days was implemented, reaching a stable temperature of 20 °C, which was maintained till the culmination of the study. The relative humidity was controlled throughout the trial to maintain a minimum variation between 60 and 70% for the first 3 days and around 50% for the rest of the trial. Continuous vigilance over the room’s temperature and ventilation was exercised throughout the study duration from day 1 to 35, with additional scrutiny at the cage-level temperature over the same period. Each enclosure, with dimensions 66 × 66 cm, was outfitted with two drinking nipples and a singular feeder. The stocking density was 100%.

For the preliminary 14 days, the chicks enjoyed unrestricted access to water and a conventional diet primarily composed of corn and soybean. Post this phase, from day 14 to 35, they were transitioned to one of four distinct dietary regimes, which were fed *ad libitum*: (1) a conventional diet anchored on corn and soybean meal (CTR); (2) a diet infused with 15% Spirulina powder (sourced from Allmicroalgae, Pataias, Portugal) (SP); (3) a diet with 15% extruded Spirulina powder (SPE); and (4) a diet boasting 15% Spirulina powder complemented with a specialized enzyme mixture, comprising 0.20% of porcine pancreatin extract (procured from Merck, Darmstadt, Germany) and 0.01% lysozyme derived from chicken egg white (sourced from SIGMA-ALDRICH, Missouri, USA) (SPM). The microalga extrusion was performed by Sparos company (Olhão, Algarve, Portugal), following detailed conditions: 340 mL of water addition per minute, at 34 bars and 118 °C for the last extrusion barrel. This procedure occurred from 3 to 7 s. Then, the algal pellets were dried for 8 and 10 min at 120 °C [17, 18]. The porcine pancreatin extract encapsulated 350 FIP-U/g of protease, 6000 FIP-U/g of lipase, and 7500 FIP-U/g of amylase. Lysozyme powder contained 70,000 U/mg protein. All diets were meticulously formulated to satiate the nutritional requisites delineated by the NRC [77] and were in the mash form. The compositions and nutrient content analyses of the starter and grower diets alongside microalga powder are delineated in Table 8. During the trial, a 2.5% mortality was observed (3 animals of 120 in total).

**Table 8:** Ingredient composition and nutrient content analysis of broiler experimental grower diets and *Arthrospira platensis* powder (day 14–35)

	Microalga			Diets^1^			
Item	*A. platensis*	Extruded *A. platensis*		CTR	SP	SPE	SPM
Ingredients, %							
Corn	-	-		55.4	62.2	63.7	62.2
Soybean meal	-	-		36.9	18.6	17.3	18.6
Sunflower oil	-	-		4.10	1.00	0.80	1.00
Sodium chloride	-	-		0.40	0.40	0.41	0.40
Calcium carbonate	-	-		1.08	1.39	1.38	1.39
Dicalcium phosphate	-	-		1.60	0.90	0.92	0.90
DL-Methionine	-	-		0.12	0.03	0.04	0.03
L-Lysine	-	-		0.0	0.05	0.10	0.05
Vitamin-mineral premix^2^	-	-		0.40	0.40	0.40	0.40
*A. platensis* powder	-	-		0.00	15.0	0.00	15.0
*A. platensis* extruded powder	-	-		0.00	0.00	15.0	0.00
Enzyme mixture	-	-		0.00	0.00	0.00	0.21
**Proximal composition** ^**3**^							
Gross energy, MJ/kg DM	18.7	18.9		19.3	18.5	18.3	18.5
Crude Protein, % DM	52.6	53.7		24.1	23.0	23.5	23.5
Crude fat, % DM	6.56	5.81		7.56	5.15	4.82	5.06
Ash, % DM	21.6	21.3		6.29	8.19	8.44	8.47
**Fatty acid profile, % total fatty acids**							
14:0	0.43	0.39		0.072	0.11	0.11	0.11
16:0	39.7	49.5		9.74	20.2	16.7	17.2
16:1c9	4.95	4.34		0.12	0.73	0.87	0.81
17:0	0.41	0.45		0.063	0.15	0.13	0.12
17:1*c*9	1.88	1.60		0.00	0.22	0.29	0.24
18:0	1.75	1.68		3.41	3.12	2.32	2.62
18:1c9	6.34	5.32		29.1	25.0	26.6	25.9
18:2n-6	17.8	15.0		52.9	43.0	45.6	45.4
18:3n-3	15.8	12.0		1.15	2.94	3.46	3.30
20:0	0.189	0.20		0.53	0.67	0.43	0.52
22:0	0.247	0.29		0.99	0.65	0.35	0.53
20:1c11	0.91	0.029		0.19	0.20	0.21	0.20
24:0	0.150	0.17		0.45	0.39	0.25	0.32
**Diterpene profile, µg/g**							
α-Tocopherol	27.2	29.5		47.5	29.1	24.4	34.7
α-Tocotrienol	n.d.	n.d.		4.59	3.52	4.12	3.80
β-Tocopherol	n.d.	n.d.		0.55	0.35	0.30	0.35
γ-Tocopherol + β-tocotrienol	n.d.	n.d.		6.44	5.59	6.47	5.86
γ-Tocotrienol	n.d.	n.d.		n.d.	n.d.	n.d.	n.d.
δ-Tocopherol	n.d.	n.d.		0.80	0.33	0.30	0.37
**Pigments, µg/g DM**							
Chlorophyll *a*^4^	866	1467		13.3	472	209	494
Chlorophyll *b*^5^	191	1189		23.5	91.7	167	81.2
Total chlorophylls^6^	1057	2656		36.8	564	376	575
β-Carotene	243	78.0		0.96	48.4	23.9	55.0
Total carotenoids^7^	243	154		3.06	117	43	126
Total chlorophylls and carotenoids^8^	1300	2810		39.8	681	419	701
**Minerals, mg/kg DM**							
***Macromineral***s							
Calcium (Ca)	42.8	32.2		13.5	16.7	16.9	17.5
Magnesium (Mg)	3.06	3.21		2.21	1.91	2.00	2.04
Phosphorous (P)	11.4	11.6		8.18	6.87	7.37	7.49
Potassium (K)	22.9	19.7		11.0	9.77	10.1	10.3
Sodium (Na)	34.2	25.7		5.55	8.85	9.60	9.41
Sulphur (S)	8.43	8.03		3.30	3.27	3.50	3.48
Total	123	100		43.7	47.4	49.5	50.3
***Microminerals***							
Copper (Cu)	0.010	0.006		0.014	0.013	0.012	0.010
Iron (Fe)	0.90	0.89		0.15	0.23	0.25	0.25
Manganese (Mn)	0.094	0.083		0.090	0.071	0.073	0.086
Zinc (Zn)	0.064	0.064		0.087	0.075	0.086	0.089
Total	1.06	1.04		0.34	0.39	0.42	0.43
Total macro and microminerals	124	101		44.0	47.8	49.9	50.7

^1^ CTR: Corn and soybean meal-based diet, in the control group; SP: 15% Spirulina; SPE: 15% extruded Spirulina; SPM: 15% Spirulina + 0.21% enzyme mixture (0.20% porcine pancreatin + 0.01% lysozyme)

^2^ Premix provided the following per kilogram of diet: pantothenic acid 10 mg, vitamin D_3_ 2400 IU, cyanocobalamin 0.02 mg, folic acid 1 mg, vitamin K_3_ 2 mg, nicotinic acid 25 mg; vitamin B_6_ 2 mg, vitamin A 10,000 UI, vitamin B_1_ 2 mg, vitamin E 30 mg, vitamin B_2_ 4 mg, Cu 8 mg, Fe 50 mg, I 0.7 mg, Mn 60 mg, Se 0.18 mg, Zn 40 mg

^3^ Nutrient content analysed

^4^ Ca: Chlorophyll a = 11.24 × A662 nm − 2.04 × A645 nm

^5^ Cb: Chlorophyll b = 20.13 × A645 nm − 4.19 × A662 nm

^6^ Ca + b: Total chlorophylls = 7.05 × A662 nm + 18.09 × A645 nm

^7^ Cx + c: Total carotenoids = (1000 × A470 nm − 1.90 × Ca − 63.14 × Cb) / 214

^8^ Ccc: Total chlorophylls and carotenoids = (Ca + b) + (Cx + c)

n.d.: Not detected

The experimental framework was structured around 10 replicate cages, each housing 3 birds. Thus, *n* = 30 (number of birds/cage x replication cages) was the sample size considered for the analysis of growth performance parameters, whereas *n* = 10 was applied for the other evaluations. Weekly assessments were conducted to record the weights of both broilers and feeders, with daily feed provisioning to compute the parameters of body weight gain (ascertained by the weekly weight differential divided by 7), average daily feed intake (calculated as weekly consumption per cage, divided by 7) and the feed conversion ratio (the quotient of weekly consumption divided by 3 and weekly body weight gain). For dietary analysis, samples were evaluated for dry matter (DM) content by subjecting them to a drying process at 103 °C until a steady weight was reached. The nitrogen (N) composition of the diets was gauged utilizing the Kjeldahl method in line with AOAC method 954.01 [78], with the crude protein content extrapolated as 6.25 times the nitrogen content. Ash content was appraised adhering to AOAC method 942.05 [78]. Crude fat analysis was undertaken by extracting feed samples with petroleum ether via an automatic Soxhlet extractor (Tecator Soxtec, Foss Iberia, Barcelona, Spain), post a preliminary hydrolysis with hydrochloric acid. The gross energy content of the feed was ascertained by employing adiabatic bomb calorimetry (Parr 1261, Parr Instrument Company, Moline, IL, USA).

On the 35th day, a bird from each cage, representative of the median weight, was subjected to electrical stunning followed by manual exsanguination. Blood samples were collected in Sarstedt tubes (Numbrecht, Germany) and centrifuged to separate the serum. The gastrointestinal (GI) organs including the crop, gizzard, duodenum, jejunum, ileum, and caecum were extracted and emptied, and the respective weights were documented. Additionally, the lengths of the duodenum, jejunum, ileum and caecum were measured. The duodenum, characterized by its “U” shape encircling the pancreas, constitutes the proximal segment of the intestinal tract. Following the duodenum is the jejunum, while the ileum is situated anterior to the caecal junction. The caecum, a paired tubular structure, is located distally along the ileum from the ileo-caecal-colic junction [79]. The viscosity of the contents within the small intestine was evaluated as per the methodology delineated by Pestana et al. [22]. In brief, samples harvested from the duodenum coupled with the jejunum and ileum were centrifuged for 10 min at 9,000 rpm, post which the viscosity of the resultant supernatant was gauged employing a viscometer (Model LVDVCP-II, Brookfield Engineering Laboratories, Middleboro, MA, USA) in a room with the temperature sustained at 24 °C. Segments of breast and thigh muscles (deboned and devoid of skin) were minced, enveloped in aluminium foil, placed in vacuum-sealed bags, and conserved at -20 °C pending subsequent analyses.

### Assessment of carcass characteristics

The evaluation of meat pH and colour was conducted by the methodologies delineated in *Pestana et al.* [22] and Alfaia et al. [75]. In essence, the right breast (*pectoralis major*) and thigh (*biceps femoris*) muscles were carefully deboned and skinned. Triplicate readings were obtained from three distinct regions on each muscle type. The pH values were ascertained using a glass penetration pH electrode (HI9025, Hanna Instruments, Woonsocket, RI, USA). Colour attributes, including lightness (L*), redness (a*), and yellowness (b*), were gauged employing a Minolta CR-300 Chromameter (Minolta Camera Co. Ltd., Osaka, Japan), which operates based on the CIELAB colour space framework. These measurements were documented post a 24-hour cooling interval post-mortem at 4 °C, succeeded by an hour of aerial exposure.

### Assessment of meat lipid oxidative stability

Around 1.5 g of minced meat from the left breast of each bird was segregated into four segments and encased in plastic bags. These segments were subsequently aerated and stored in a freezer maintained at 4 °C for 0 and 8 days. The level of thiobarbituric acid (TBA) reactive substances, indicative of lipid oxidation, was evaluated on both day 0 and day 8. Adhering to the spectrophotometric procedure delineated by Mercier et al. [80], the capacity of malondialdehyde, a lipid oxidation product, to engender a pink-hued chromogen that absorbs light at 532 nm was analysed. The assessments were conducted employing a UV/visible spectrophotometer (Genesys 150, ThermoScientific, Madison, USA). For TBA reactive substances quantification, a standard calibration curve was generated using 1,1,3,3-tetraethoxypropane (Fluka, Neu Ulm, Germany) as a malondialdehyde precursor. The outcomes are articulated as milligrams of malondialdehyde per kilogram of meat.

### Analysis of total cholesterol, diterpenes, pigments and minerals in meat and experimental diets

The quantification of total cholesterol, β-carotene, and tocopherols in both fresh meat (750 mg) and feed (100 mg) was conducted following the methodology elucidated by Prates et al. [81], with additional particulars furnished in Alfaia et al. [75], Pestana et al. [22] and Costa et al. [76]. Succinctly, samples were submitted to direct saponification and, then, an aliquot of n-hexane layer was filtered and injected into an HPLC system (Agilent 1100 Series; Agilent Technologies Inc., Palo Alto, CA) incorporated with a normal phase silica column (250 mm x 4.6 mm i.d., 5-µm particle size; Zorbax RX-Sil, Agilent Technologies Inc., Palo Alto, CA, USA) coupled, in series, with a fluorescence detector of diterpenes (excitation at λ = 295 nm and emission at λ = 325 nm) and a UV-visible photodiode array detector of β-carotene (λ = 450 nm) and cholesterol (λ = 202 nm). Duplicate measurements were carried out, and concentrations were ascertained utilizing the external standard technique, derived from a standard curve correlating peak area and concentration.

For the analysis of chlorophyll *a*, chlorophyll *b* and total carotenoids, the technique delineated by Teimouri et al. [82] was adopted, with minor alterations detailed in Alfaia et al. [65], Pestana et al. [22] and Costa et al. [76]. All operations concerning pigment extraction and evaluation were executed under subdued lighting conditions to curtail the photodegradation of pigments. The pigment contents were calculated using formulae provided by Hynstova et al. [15].

The assessment of the mineral profile, encompassing calcium (Ca), potassium (K), magnesium (Mg), sodium (Na), phosphorus (P), sulphur (S), copper (Cu), iron (Fe), manganese (Mn), and zinc (Zn), adhered to the procedures described in Ribeiro et al. [83] and Costa et al. [76]. Briefly, ground samples (300 mg) were dissolved, for 16 h, in 3 mL of concentrated nitric acid and 10 mL of hydrochloric acid added to each digestion tube. Then, 1 mL of hydrogen peroxide was added to avoid sample loss and the tubes were randomly distributed in a digestion plate (DigiPREP MS, SCP Science, Quebec, Canada). The mineralization occurred according to the following pattern: 1 h to reach 95 °C and 1 h at 95 °C. Afterwards, samples were cooled in a ventilated chamber, diluted with distilled water, and filtered with 90-mm diameter filter papers (Filtros Anoia S.A., Barcelona, Spain). Inductively Coupled Plasma Optical Emission Spectrometry (ICP-OES) employing an iCAP 7200 duo instrument (Thermo Scientific, Waltham, MA, USA) was utilized for the analysis. Calibration curves generated from multi-element standards (PlasmaQual S22, SPC Science, Baie-D’Urfé, QC, Canada) were deployed to quantify the distinct elements.

### Assessment of dry matter and total lipid in meat

The quantification of dry matter in breast and thigh meats was conducted by the methodology delineated by Rosenkranz [84]. Samples were freeze-dried utilizing a freeze-dryer (Labogene, CoolSafe, Lillerod, Denmark) operated at -60 °C and 2.0 hPa. The freeze-drying process took place for at least 72 h, always making sure that the weight of the dried samples was constant. Post freeze-drying, the specimens were preserved in desiccators at ambient temperature pending further evaluation.

For the assessment of total lipids in both meat samples and feed, the freeze-dried breast and thigh muscles underwent lipid extraction as per the procedure articulated by Folch et al. [85]. The extraction was executed employing a blend of dichloromethane and methanol at a volumetric ratio of 2:1. Subsequently, the extracted lipids were evaporated to dryness, with the resultant fatty residue being weighed gravimetrically. The measurements were conducted in duplicate.

### Analysis of fatty acid profile in meat and experimental diets

The assessment of fatty acid composition in breast and thigh muscles was performed by transforming the fatty acid residue into fatty acid methyl esters (FAME). This transformation employed a dual-stage transesterification process comprising basic and acidic steps. Initially, the fatty acids underwent transesterification using NaOH dissolved in anhydrous methanol (0.5 M) at a temperature of 50 °C for 30 min. This was followed by a secondary transesterification step employing an HCl/methanol mixture (1:1 v/v) at 50 °C for 10 min, as outlined by Raes et al. [86]. A similar procedure was applied to the analysis of FAME in feed samples, albeit with a direct transesterification using HCl/methanol (1:1 v/v) at 70 °C for 2 h.

The analytical procedure for FAME was carried out by the methodology detailed by Pestana et al. [22]. The analysis utilized a Gas Chromatography system (GC System, 7890 A, Agilent Technologies, California, USA) equipped with a Supelcowax® 10 capillary column (30 m × 0.20 mm internal diameter, 0.20 μm film thickness; Supelco, Bellefonte, PA, USA) and a flame ionization detector, under specified parameters. Both the injector and detector temperatures were configured at 250 and 280 °C, respectively. Helium was employed as the carrier gas with a flow rate of 1.0 mL/min, and a split ratio of 1:20. The temperature protocol for the gas chromatograph oven commenced with an initial temperature of 50 ºC held for 4 min, then increased at a rate of 13 °C/min to 175 °C (sustained for 20 min), followed by a rate of 4 °C/min to 275 °C (sustained for 44 min).

The identification of FAME was facilitated through the comparison of retention times against a standard reference (FAME mix 37 components, Supelco Inc., Bellefonte, PA, USA), which was further verified by Gas Chromatography-Mass Spectrometry (GC-MS QP2010-Plus, Shimadzu, Kyoto, Japan). The quantification of FAME was conducted using heneicosanoic acid (21:0) methyl ester as the internal standard. The derived fatty acid composition data was expressed in terms of grams per 100 g of total fatty acids.

### Calculation of health-related lipid indices

Health-related lipid indices were calculated according to Ulbricht & Southgate [87]and Erickson [88], using fatty acids profile, and following specific formulas: AI = [C 12:0 + (4 × C 14:0) + C 16:0]/Σ UFA [87]; TI = (C 14:0 + C 16:0 + C 18:0)/[((0.5 × ΣMUFA) + (0.5 × ΣFAω6) + (3 × ΣFAω3))+ ( ΣFAω3/ ΣFAω6)] [87]; PI = (monoenoic acid × 0.025) + (dienoic acid × 1) + (trienoic acid × 2) + (tetraenoic acid × 4) + (pentanoic acid × 6) + (hexanoic acid × 8) [88].

### Data analysis

The data collected were subjected to analysis employing the Generalized Linear Mixed (GLM) model within the SAS software environment (SAS Institute Inc., Cary, NC, USA) for the majority of the variables. An exception was made for the TBA reactive substances values, for which the Repeated Measures (PROC MIXED) procedure within SAS was utilized due to the time-dependent nature of the measurements. The experimental unit was the cage for variables such as body weight, body weight gain, average daily feed intake and feed conversion ratio, while the bird was the experimental unit for metrics including nutritional assessments, such as gastro-intestinal length and weight and gut content viscosity, and also meat quality variables.

A multiple comparisons test was conducted using the PDIFF option to discern statistical variances among the dietary interventions. Adjustments were made employing the Tukey-Kramer method to control for multiple comparison errors. The threshold for statistical significance was set at a *p*-value of less than 0.05 to identify meaningful distinctions among the groups.

## Data Availability

No datasets were generated or analysed during the current study.

## Abbreviations

ADFI: Average daily feed intake
ADG: Average daily gain
AI: Atherogenic Index
CAZymes: Carbohydrate-active enzymes
CTR: Control group
DHA: Docosahexaenoic acid
DPA: Docosapentaenoic acid
EPA: Eicosapentaenoic acid
FAME: Fatty acid methyl esters
FCR: Feed conversion ratio
GC-MS: Gas chromatography-mass spectrometry
ICP-OES: Inductively coupled plasma optical emission spectrometry
LC-PUFA: Long-chain polyunsaturated fatty acids
MUFA: Monounsaturated fatty acids
PI: Peroxidisability index
PUFA: Polyunsaturated fatty acids
SFA: Saturated fatty acids
SP: Spirulina group
SPE: Extruded Spirulina group
SPM: Spirulina with enzyme mixture group
TBARS: Thiobarbituric acid reactive substances
TI: Thrombogenic Index
